# Chronic kidney disease in Kuwait: a multicenter study of two cohorts with different levels of access to public healthcare

**DOI:** 10.1186/s12882-024-03794-6

**Published:** 2024-10-16

**Authors:** Ali AlSahow, Anas AlYousef, Nasser AlSabti, Bassam AlHelal, Heba AlRajab, Ahmed AlQallaf, Yousif Bahbahani, Abdulrahman AlKandari, Ahmad Mazroue, Noha Dewidar, Gamal Nessim, Ahmad Atef Mekky, Mohamed Sherif, Hesham Zamel, Ahmed Abdalla, Rajeev Kumar

**Affiliations:** 1 Division of Nephrology, Jahra Hospital, Jahra Central, PO Box 2675, 01028 Jahra, Kuwait; 2 Division of Nephrology, Amiri Hospital, Kuwait City, Kuwait; 3 Division of Nephrology, Mubarak Hospital, Jabriya, Kuwait; 4 Division of Nephrology, Adan Hospital, Hadiya, Kuwait; 5 Division of Nephrology, Farwaniya Hospital, Sabah Al Nasser, Kuwait; 6 Division of Nephrology, Jaber Hospital, Kuwait, Kuwait; 7 Division of Nephrology, Sabah Hospital, Kuwait, Kuwait; 8 BRA IRCH, All India Institute of Medical Sciences, Delhi, India

**Keywords:** Chronic kidney disease, Diabetes mellitus, Hypertension, Healthcare access, Kuwait

## Abstract

**Introduction:**

Kuwait has a large expatriate community who experience both restricted access to public health services and lower income than Kuwaiti citizens. Given these conditions, we examined differences in characteristics and management of chronic kidney disease (CKD) between Kuwaitis and expatriates.

**Methods:**

Clinical and laboratory data for adult CKD Stages 3–5 not on dialysis (CKD 3–5 ND) patients with native kidneys attending nephrology clinics in all Ministry of Health hospitals collected from January 1, 2022, to December 31, 2022. Cohort was then divided into Kuwaiti patients and expatriates patients for comparison.

**Results:**

We collected data from 2,610 patients (eGFR: 30.8 ml/min/1.73m^2^; age: 62.6 years; males: 56.7%; Kuwaitis: 62.1%). Kuwaitis were older (63.94 vs. 60.3 years, *p* < 0.001), with lower mean eGFR (30.4 vs. 31.5 ml/min/1.73m^2^, *p* = 0.052) than non-Kuwaitis, however, Kuwaitis had lower mean blood pressure (137.2/76.5 vs. 139.1/78.9 mmHg, *p* = 0.006), lower HbA1c in diabetics (7.59 vs. 7.82%, *p* = 0.010), and better lipid profile despite higher body mass indexes (29.6 vs. 28.9 kg/m^2^, *p* = 0.002). Both groups had high diabetes mellitus and hypertension rates. Sodium-glucose cotransporter 2 inhibitors (SGLT2i) were used in only 22.6% and renin-angiotensin-aldosterone system inhibitors (RAASi) in only 46.2%.

**Conclusion:**

CKD 3–5 ND is caused by diabetes mellitus in 56.6% of cases, and the majority have hypertension. In our study, non-Kuwaitis had higher eGFR; however, restricted public healthcare access and lower income can lead to an unhealthy diet and suboptimal care, which may cause higher blood pressure, higher HbA1c, and a higher dyslipidemia rate. RAASi and SGLT2i utilization must increase to combat CKD, and antihypertensive selection must improve.

## Introduction

The global prevalence of chronic kidney disease (CKD) is 9.1% [1] and is expected to rise due to increasing prevalences of diabetes mellitus and obesity, population aging, and polypharmacy [2]. Despite its high prevalence and individuals’ increased risk of cardiovascular disease (CVD) and all-cause and cardiovascular mortality [3], less than 50% of individuals with CKD not on dialysis were aware of their condition [4]. Kuwait is plagued with high rates of diabetes mellitus, the leading cause of CKD worldwide [5, 6], hypertension (HTN), obesity, smoking, and sedentary lifestyles [7, 8], all of which are risk factors for the initiation and progression of CKD. However, little is known about the prevalence of advanced CKD, its causes, management, and complications in Kuwait.

Kuwait has a large ethnically diverse non-Kuwaiti (expatriate) community, representing 66% of the total population of 4,385,717 in 2021 [9]. The Ministry of Health (MoH) provides the majority of healthcare services to the population of Kuwait. Kuwaitis have full free access to all MoH outpatient and inpatient services with laboratory and radiology services as well as free prescriptions. Expatriates, on the other hand, must pay for an annual MoH insurance card to access MoH services. Other applicable fees paid upfront include fees for emergency department or outpatient clinic visits (which cover the cost of basic blood tests and the prescription of certain medications). Admission to MoH hospitals for expatriates, whether urgent or elective is unrestricted, and inpatient services, including surgical interventions, vascular access procedures, preoperative evaluation (anesthesia, cardiology, etc.), and acute dialysis (intermittent or continuous) are provided free of charge. However, expatriates must pay for any hospitalization stay and inpatient radiology services and kidney biopsies are provided at a subsidized fee through a charity group [10].

To complicate matters, most expatriates earn a lower income than Kuwaitis and cannot afford private healthcare due to its prohibitive cost. Given their lower income, many expatriates are forced into substandard housing conditions and consumption of cheap unhealthy food. The majority are also working more physically demanding jobs at longer hours. Furthermore, many expatriates elect to visit public primary care clinics instead of hospitals due to the lower cost per visit, mainly to refill permissible medications and complete blood tests. However, the lack of services and proper follow-up provided by clinics could impact patient outcomes [10]. Research has shown that low income and poor access to healthcare increase the risk of kidney disease [11]. In this paper, we examined the prevalence and management of CKD Stages 3–5 not on dialysis (CKD 3–5 ND) in Kuwait with a focus on the characteristics and management of CKD between Kuwaiti patients, who have higher incomes and better access to public healthcare, and expatriates, who have lower incomes and restricted access to public healthcare.

## Methods

We reviewed the medical records of all patients with CKD 3–5 ND above the age of 12 years with native kidneys attending adult nephrology clinics in all seven hospitals of the MoH in Kuwait from January 1, 2022, to December 31, 2022. We recruited patients aged 12 years and above because this is the age children are transferred to adult care in Kuwait. We recorded demographics (age, nationality, sex, occupation, marital status, etc.), baseline estimated glomerular filtration rate (eGFR), and CKD stage according to the Kidney Disease Improving Global Outcome (KDIGO) guidelines [12] at the time of recruitment. We also gathered data relating to the cause of CKD, comorbidities, laboratory results, and medications for each participant. eGFR was calculated using the creatinine-based Chronic Kidney Disease Epidemiology Collaboration (CKD-EPI) formula [13]. The cohort was then divided into Kuwaiti and non-Kuwaiti groups to compare characteristics and management.

Data analysis was performed using the statistical software Stata16 (StataCorp, College Station, TX, USA). Data were summarized using descriptive statistics (mean and standard deviation) for continuous variables and frequency for categorical variables. We applied the bivariable analysis student-t test and chi-square/Fisher’s exact test to compare the two subgroups. We included the plausible variables (demographical, CKD cause, management) in the multivariable logistic regression to identify variables differentiating the two groups. We calculated the effect size using Cohen’s d for laboratory variables to determine the clinical importance. A p-value of less than 0.05 was considered significant.

## Results

Table 1 shows the basic characteristics of the total cohort and the two subgroups. The total number of patients with CKD 3–5 ND recruited from all nephrology outpatient clinics in all seven MoH hospitals was 2,610 (mean eGFR: 30.8 ml/min/1.73m^2^; mean age: 62.6 years; males: 56.7%; above the age of 65: 47.0%; Kuwaitis: 62.1%). Compared with the expatriate group, the Kuwaiti group was older (63.94 vs. 60.3 years for expatriates, *p* < 0.001), had a greater number of participants above the age of 65 (54.0% vs. 35.6% for expatriates, *p* < 0.001), and fewer men (49.8% vs. 67.9% for expatriates, *p* ≤ 0.001). Kuwaitis also had lower mean eGFR, although this was statistically insignificant (30.4 vs. 31.5 ml/min/1.73m^2^ for expatriates, *p* = 0.052). Blood pressure (BP) was lower in Kuwaiti patients (137.2/76.5 vs. 139.1/78.6 mmHg for expatriates, *p* = 0.006 for systolic and < 0.001 for diastolic); however, body mass index (BMI) was higher for Kuwaitis (29.6 vs. 28.9 kg/m^2^ for expatriates, *p* = 0.002). The two groups had similar rates of DM, HTN, coronary artery disease, obesity, and smoking. However, diabetic kidney disease, and hypertensive kidney disease, as causes of CKD were higher in Kuwaiti patients (DM: 59.3% vs. 52.0% for expatriates, *p* < 0.001, and HTN: 20.5% vs. 15.6%, *p* = 0.001). Socioeconomic information was obtained in less than two thirds of the cohort. A higher percentage of expatriates were married (88.8% vs. 82.8% of Kuwaitis, *p* = 0.001), fewer were unemployed/retired (38.5% vs. 50.7% of Kuwaitis, *p* = 0.002), and they accounted for all the manual labor workers in the sample. No difference emerged between the two groups in terms of education; however, illiteracy was significantly higher in older Kuwaiti females accounting for 72.7% of all female illiteracy. Table 2 details the contribution from each hospital, and differences in patients’ characteristics, if any between the participating hospitals. The size of the catchment area played a role in the number of recruited patients. In addition, Jahra, Farwaniya, and Mubarak areas are favored by expatriates, which explains their higher percentages in the corresponding hospitals. furthermore, Jaber Hospital, which opened in 2022, only treats Kuwaiti patients compared to other public hospitals.

**Table 1:** Basic baseline characteristics of patients with CKD 3–5 not on dialysis at recruitment

	Total cohort 2,610	Kuwaitis 1,622 (62.1%)	Non-Kuwaitis 988 (37.9%)	*p*-value
Age, year, mean ± SD	62.6 ± 15.2	63.94 ± 15.57	60.3 ± 14.24	< 0.001
Male sex, n (%)	1479 (56.7%)	808 (49.8%)	671 (67.9%)	< 0.001
eGFR, ml/min/1.73m^2^, mean ± SD	30.8 ± 14.42	30.4 ± 14.40	31.5 ± 14.44	0.052
Systolic BP, mm/Hg, n, mean ± SD *	2544 137.9 ± 16.6	1593 137.2 ± 15.8	951 139.1 ± 177	0.006
Diastolic BP, mm/Hg, n, mean ± SD *	2544 77.3 ± 10.2	1593 76.5 ± 10.2	951 78.6 ± 10.0	< 0.001
BMI, kg/m^2^, n, mean ± SD	1991 29.3 ± 5.2	1284 29.6 ± 5.21	707 28.9 ± 5.2	0.002
Age > 65 year, n (%)	1228 (47.0%)	876 (54.0%)	352 (35.6%)	< 0.001
**Comorbidities**, n (%) ** DM HTN CAD BMI > 30 PAD Smoking/COPD CVA Autoimmune disorders Urinary tract disorders *** History of AKI in the past 12 months Cancer Gastrointestinal and liver diseases No comorbidity	1782 (68.3%) 2321 (88.9%) 954 (36.6%) 722 (27.7%) 110 (4.2%) 369 (14.1%) 150 (5.8%) 124 (4.8) 338 (13.0%) 177 (6.8%) 95 (3.6%) 116 (4.4%) 54 (2.1%)	1132 (69.8%) 1449 (89.3%) 593 (36.6%) 466 (28.7%) 71 (4.4%) 219 (13.5%) 100 (6.2%) 89 (5.5%) 173 (10.7%) 95 (5.9%) 54 (3.3%) 87 (5.3%) 35 (2.2%)	650 (65.8%) 872 (88.3%) 361 (36.5%) 256 (25.9%) 39 (3.9%) 150 (15.2%) 50 (5.1%) 35 (3.5%) 165 (16.3%) 82 (8.3%) 41 (4.1%) 29 (2.9%) 19 (1.9%)	0.033 0.396 0.991 0.118 0.596 0.232 0.240 0.024 < 0.001 0.016 0.278 0.004 0.683
**Cause of CKD**, n (%) ** DKD HTN-Vasculopathy Glomerulopathies PCKD Others Unknown	1476 (56.6%) 486 (18.6%) 232 (8.9%) 78 (3.0%) 303 (11.6%) 255 (9.6%)	996 (59.3%) 332 (20.5%) 140 (8.6%) 41 (2.4%) 192 (11.4%) 131 (8.1%)	514 (52.0%) 154 (15.6%) 92 (9.3%) 37 (3.7%) 114 (11.5%) 124 (12.6%)	< 0.001 0.001 0.554 0.077 0.317 < 0.001
**Education**,** n (%) #** Illiterate School education Post High School Education	**1**,**552** 569 (36.7%) 572 (36.9%) 411 (26.5%)	**935** 335 (35.8%) 346 (37.0%) 254 (27.2%)	**617** 234 (37.9%) 226 (36.6%) 157 (25.4%)	0.069 0.355 0.875
**Marital status**,** n (%) #** Single Married with or without children Divorced or Widowed	**1**,**583** 91 (5.7%) 1367 (86.4%) 125 (7.9%)	**967** 60 (6.2%) 801 (82.8%) 96 (9.9%)	**626** 31 (5.0%) 556 (88.8%) 29 (4.6%)	0.448 0.001 0.001
**Employment**,** n (%) #** Student Unemployed Retired Manual labor Office job/ Clerk Firefighter-Army-Police-National Guard Professional (Dr, teacher, engineer, lawyer, etc.)	**1**,**558** 37 (2.4%) 715 (45.9%) 329 (21.1%) 138 (8.9%) 262 (16.8%) 15 (1.0%) 61 (3.9%)	**942** 23 (2.4%) 478 (50.7%) 265 (28.1%) 0 (0.0%) 130 (13.9%) 11 (1.2%) 34 (3.6%)	**616** 14 (2.3%) 237 (38.5%) 64 (10.4%) 138 (22.0%) 132 (21.4%) 4 (0.6%) 27 (4.4%)	0.998 0.002 0.001 < 0.001 0.001 0.370 0.298

Values are given as mean ± SD for continuous variables and as number (percentage) for categorical variables

* Data on each of these variables was not available for all patients

** More than 1 answer per patient was permissible, that explains why total % is greater than 100%

*** Urinary tract disorders: Calculi, reflux, infection, obstruction

# Data on education, marital status, and employment was only available in the number of cases shown in bold

eGFR, estimated glomerular filtration rate; BP, blood pressure; DM, diabetes mellitus; HTN, hypertension; CAD, coronary artery disease; BMI, body mass index; PAD, peripheral arterial disease; COPD, chronic obstructive pulmonary disease, CVA, cerebrovascular accident; AKI, acute kidney injury; CKD, chronic kidney disease; DKD, diabetic kidney disease; PCKD, polycystic kidney disease

**Table 2:** Contribution from each of all seven ministry of health hospitals

	Adan	Amiri	Farwaniya	Jahra	Jaber *	Mubarak	Sabah
Total	297	508	368	397	110	706	224
Mean age, years, ± SD	61.84 ± 14.1	62.07 ± 15.0	59.95 ± 14.1	60.1 ± 15.7	64.3 ± 14.2	65.4 ± 14.4	63.3 ± 16.5
Mean eGFR, ml/min/1.73m^2^, ± SD	33.38 ± 14.4	26.62 ± 14.1	31.22 ± 13.8	30.6 ± 14.85	34.82 ± 13.98	32.38 ± 14.3	29.31 ± 14.0
Male, n (%)	135 (45.5%)	302 (59.4%)	228 (62.0%)	198 (49.9%)	67 (60.9%)	422 (59.8%)	127 (56.7%)
Kuwaitis, n (%)	275 (92.6%)	382 (75.2%)	127 (34.5%)	197 (49.6%)	110 (100.0%)	378 (53.5%)	153 (68.3%)
Diabetics, n (%)	208 (70.0%)	343 (67.5%)	243 (66.0%)	81 (73.6%)	251 (63.2%)	478 (67.7%)	178 (79.5%)

* Jaber hospital caters for Kuwaiti patients only

eGFR, estimated glomerular filtration rate

Tables 3, 4 details the laboratory results of the cohort at the time of recruitment. HbA1c (in diabetics) was lower in Kuwaitis (7.59 vs. 7.82% for expatriates, *p* = 0.01). Parathyroid hormone was slightly higher in Kuwaitis (17.6 vs. 16.0 pmol/L for expatriates, *p* = 0.023) with higher phosphate (1.34 vs. 1.29 for expatriates, *p* ≤ 0.001) but similar calcium values. Kuwaitis also had a better lipid profile. Hemoglobin levels were significantly lower in Kuwaitis (119.2 vs. 124.1 g/L for expatriates, *p* < 0.001) but no difference was evident between iron profiles. Potassium levels were similar in both groups, even in patients on renin-angiotensin-aldosterone system inhibitors (RAASi); however, potassium was higher in diabetics (4.61 vs. 4.5 mmol in nondiabetics, *p* < 0.001). Uric acid levels were within the normal range with no difference between the two groups.

**Table 3:** Laboratory values for patients with CKD 3–5 not on dialysis at recruitment

	Total available *	Kuwaitis	Non-Kuwaitis	*p*-value	Cohen’s d effect size
Hgb, (g/L), n, mean ± SD	2610 121.1 ± 19.8	1622 119.2 19.3	988 124.1 ± 20.3	< 0.001	0.247
Iron, mmol/L, n, mean ± SD	2020 12.2 ± 5.4	1360 12.1 ± 5.4	660 12.3 ± 5.2	0.374	0.038
Transferrin saturation %, n, mean ± SD	2139 22.2 ± 9.8	1436 22.4 ± 10.2	703 21.9 ± 9.0	0.255	0.052
Ferritin, ng/mL, n, mean [25th -75th percentile)	558 163 [83.5-230.8]	363 170 [77–240]	195 151 [88–220]	0.456	0.082
HbA1c (in 1847 diabetics), %, n, mean ± SD	1584 7.67 ± 1.68	1033 7.59 ± 1.62	551 7.82 ± 1.79	0.010	0.135
Potassium, mmol/L, n, mean ± SD	2609 4.6 ± 0.56	1622 4.6 ± 0.56	899 4.6 ± 0.55	0.237	0.054
HCO_3_, mmol/L, n, mean ± SD	2299 23.7 ± 3.8	1334 23.7 ± 3.8	955 23.8 ± 3.7	0.747	0.027
Uric Acid, µmol/L, n, mean ± SD	2581 412 ± 412.3	1608 414.2 ± 106	973 409.2 ± 107	0.250	0.047
Corrected Calcium, mmol/L, n, mean ± SD	2552 2.33 ± 0.16	1614 2.33 ± 0.16	938 2.33 ± 0.14	0.633	0.019
Phosphate, mmol/L, n, mean ± SD	2591 1.32 ± 0.32	1612 1.34 ± 0.354	979 1.29 ± 0.29	< 0.001	0.154
PTH, pmol/L, n, mean [25th -75th percentile]	1428 17.0[9.5–23.0]	959 17.6[10–34.0]	469 16.0[8.8–28.0]	0.023	0.104
Albumin, g/L, n, mean ± SD	2578 37.3 ± 5.3	1618 37.2 ± 5.2	960 37.4 ± 5.4	0.294	0.038
Total cholesterol, mmol/L, n, mean ± SD	2505 4.3 ± 1.2	1579 4.2 ± 1.2	926 4.4 ± 1.2	0.008	0.167
LDL, mmol/L, n, mean ± SD	2332 2.35 ± 1.0	1462 2.3 ± 1.00	870 2.5 ± 1.05	< 0.001	0.195
HDL, mmol/L, n, mean ± SD	2366 1.14 ± 0.4	1484 1.18 ± 0.41	882 1.08 ± 0.31	< 0.001	0.275
Triglycerides mmol/L, n, mean ± SD	2505 1.75 ± 1.0	1579 1.69 ± 1.0	926 1.83 ± 1.11	0.001	0.135

Values are given as mean ± SD for continuous variables and as number (percentage) for categorical variables

* Data on each of these variables was not available for all patients

BP, blood pressure; BMI, body mass index; Hgb, hemoglobin; PTH, parathyroid hormone; LDL, low density lipoprotein; HDL, high density lipoprotein

**Table 4:** Medical management of patients with CKD 3–5 not on dialysis at recruitment

	Total cohort 2610	Kuwaitis 1622 (62.1%)	Non-Kuwaitis 988 (37.9%)	*p*-value
**Aspirin**,** n (%)**	1318 (50.5%)	798 (49.2%)	520 (52.6%)	0.089
**Anti-dyslipidemia**,** n (%)** Statin Evolocumab None	2003 (76.7%) 326 (8.7%) 381 (14.6%)	1235 (76.1%) 201 (12.4%) 186 (11.5%)	768 (77.7%) 25 (2.5%) 195 (19.5%)	< 0.001
**Sodium bicarbonate**,** n (%)**	512 (19.6%)	296 (18.2%)	216 (21.9%)	0.024
**Anti-hyperuricemia**,** n (%)** Allopurinol Febuxostat None	524 (20.1%) 510 (19.5%) 1576 (60.4%)	309 (19.1%) 294 (18.1%) 1019 (62.8%)	215 (21.8%) 216 (21.9%) 557 (56.4%)	0.004
**Anti-hyperglycemics for diabetics only**,** n (%)** Insulin Metformin Sulfonylurea SGLT2i DPP4i GLP1RA	**1782** 1075 (60.3%) 345 (19.4%) 311 (17.5%) 402 (22.6%) 455 (25.5%) 56 (3.1%)	**1132** 699 (61.7%) 208 (18.4%) 148 (13.1%) 305 (26.9%) 746 (33.0%) 47 (4.2%)	**650** 376 (57.8%) 137 (21.1%) 163 (25.1%) 97 (14.9%) 81 (12.5%) 9 (1.4%)	0.105 0.165 < 0.001 < 0.001 < 0.001 0.001
**Antihypertensives**,** n (%)** RAASi Beta blockers Thiazide diuretics Potassium sparing diuretics Loop diuretics Dihydropyridine CCB Non-dihydropyridine CCB Vasodilators (hydralazine, minoxidil) Central agents (methyldopa, moxinidine)) Alpha blocker-combined alpha beta blocker	1207 (46.2%) 1165 (44.6%) 281 (10.8%) 101 (3.9%) 740 (28.4%) 1309 (50.2%) 241 (9.2%) 615 (23.6%) 1584 (60.7%) 339 (13.0%)	781 (48.2%) 683 (42.1%) 183 (11.3%) 73 (4.5%) 465 (28.7%) 924 (57.0%) 106 (6.5%) 306 (18.9%) 1023 (63.0%) 153 (9.4%)	426 (43.1%) 482 (48.8%) 98 (9.9%) 28 (2.8%) 275 (27.8%) 385(39.0%) 135 (13.7%) 309 (31.3%) 561 (56.8%) 186 (18.8%)	0.012 0.001 0.276 0.032 0.646 < 0.001 < 0.001 < 0.001 < 0.001 < 0.001
**Anti-secondary hyperparathyroidism**,** n (%)** Vitamin D analogues Calcimimetics	761 (29.2%) 77 (2.9%)	526 (32.4%) 65 (4.0%)	235 (23.8%) 12 (1.2%)	< 0.001 < 0.001
**Anti-hyperphosphatemia**,** n (%)** Calcium carbonate Sevelamer carbonate	840 (32.2%) 401 (15.4%)	532 (32.8%) 290 (17.9%)	308 (31.2%) 111 (11.2%)	0.389 < 0.001

SGLT2i, sodium glucose cotransporter 2 inhibitor; DPP4i; dipeptidyl peptidase 4 inhibitor; GLP1RA, glucagon-like peptide-1 agonists; RAASi, renin angiotensin aldosterone inhibitors; CCB, calcium channel blockers

Table 5 describes the medical management of our patients. Half of the sample (50.5%) were taking aspirin, 39.6% were receiving anti-hyperuricemia therapy (allopurinol 20.1% vs. febuxostat 19.5%), and more than 85% were on anti-dyslipidemia agents. For the management of DM, insulin was prescribed in 60.3% of the patients, while metformin was used by less than 20%, with no difference between the two groups. Sodium-glucose cotransporter 2 inhibitors (SGLT2i) were used in only 22.6% of patients (76% were Kuwaitis). For the management of HTN, centrally acting agents and dihydropyridine calcium channel blockers (CCB) were the most frequently used agents (CCB: 50.2%; central agents: 60.7%), and both were used more in Kuwaitis, followed by RAASi and beta blockers. RAASi were used in only 46.2% of the CKD 3–5 ND cohort. Alpha/combined alpha and beta blockers, non-dihydropyridine CCB, and vasodilators were used more frequently in non-Kuwaitis. In the management of mineral bone disorders (CKD-MBD), calcimimetics and sevelamer (the only non-calcium-based binder available in Kuwait in 2022) were used significantly more frequently in Kuwaitis (vitamin D analogs: 32.4% vs. 23.8% for expatriates, *p* < 0.001). Multivariable logistic regression results in Table 4 indicate that Kuwaitis with CKD 3–5 ND were older with better high-density lipoprotein profiles but higher rates of diabetic kidney disease than expatriates.

**Table 5:** Multivariable logistic regression for differentiable factors between Kuwaiti and non-kuwaiti patients with CKD 3–5 not on dialysis

Variable	Ref	Comparative Category	B(SE)	Odds ratio (95% CI)	*p*-value
Age	< 65 year	>=65 year	0.671 (0.096)	1.96 [1.62 to 2.36]	< 0.001
Sex	Male	Female	0.530 (0.099)	1.70 [1.40 to 2.06]	< 0.001
Diabetes	No	Yes	-0.170 (0.145)	0.84 [0.64 to 1.12]	0.243
Hypertension	No	Yes	-0.137 (0.148)	0.87 [0.65 to 1.17]	0.355
UTO	No	Yes	-0.482 (0.133)	0.62 [0.48 to 0.80]	< 0.001
DKD	No	Yes	0.346 (0.136)	1.42 [1.08 to 1.85]	0.011
Hypertensive disease	No	Yes	0.315 (0.125)	1.37 [1.07 to 1.75]	0.012
Hgb	continuous		-0.004 (0.002)	0.996 [0.99 to 1.0]	0.067
TC	< 5.17	>=5.17	-0.242 (0.110)	0.79 [0.63 to 0.98]	0.028
HDL	> 1.16	<=1.16	0.638 (0.169)	1.89 [1.36 to 2.64]	< 0.001

*N* = 2364, missing 246, primarily due to missing TC & HDL values

Hosmer and Lemeshow goodness of fit *p* = 0.647

UTO, urinary tract obstruction; DKD, diabetic kidney disease; Hgb, hemoglobin; TC, total cholesterol; HDL, high density lipoprotein

## Discussion

With this study, we examined the characteristics and the management of CKD by nephrologists in Kuwait to understand the prevalence and management of CKD in Kuwait and the potential impact of limited access to public healthcare services and limited income on the management of CKD and associated diseases, such as DM and HTN. According to reported literature, the age-standardized global prevalence of CKD Stages 3–5 among adults aged ≥ 20 years is 4.7% in men and 5.8% in women [14]. However, our cohort of CKD 3–5 ND under nephrology care represents only 0.06% of the total population of the country, or 0.1% of the Kuwaiti section of the total population according to the latest census [9]. Although some CKD patients are followed in the private sector, their number is small and often limited to Kuwaitis due to its high cost. Another group could be under the care of primary care physicians or internists, but they usually prefer to refer CKD patients with eGFR < 60 ml/min/1.73m^2^ to nephrology. Therefore, the lower-than-expected number of patients with CKD 3–5 ND under nephrology care could be due to a lack of awareness. A systematic review of studies estimating CKD awareness showed low awareness with pooled CKD awareness of only 19.2%, with most studies reporting < 50% of individuals with CKD being aware of their condition [4]. The higher proportion of men in the expatriates group is consistent with the fact that men account for 66% of the non-Kuwaiti population [9].

Risk factors for CKD development and progression like DM, HTN, and obesity are quite prevalent in Kuwait [6–8]. Type 2 DM, the leading cause of CKD worldwide, affects one in four Kuwaiti adults [6]. Kuwait is a high-income country with a free and accessible public healthcare system for its citizens with no restriction to any medication, laboratory tests, or imaging procedures. Given the high-income status of the country, most medications are readily available. In addition, Kuwaiti retirees also have free medical insurance giving them free access to many private sector services. However, access to this public healthcare is limited for most non-Kuwaitis, who also lack private medical insurance due to its prohibitive cost, which hinders appropriate care in this group of patients. It is well known that populations with low income and poor access to healthcare are at the highest risk for kidney disease and its complications [11]. Expatriates are screened medically for chronic diseases (DM, HTN, CKD) and infectious diseases (hepatitis, tuberculosis) before they are granted residency, which means they arrive in good health. However, with aging and exposure to environmental, dietary, and occupational factors, they develop chronic diseases, such as DM, HTN, obesity, coronary artery disease, and CKD, and need healthcare. Expatriates have to not only pay for their clinic visits (which covers the cost of basic blood tests), they must pay extra for certain tests (e.g., parathyroid hormone, ferritin) [10]. Such fees cover the cost of certain medications from the hospital pharmacy, for example, angiotensin-converting enzyme inhibitors (ACEi) but not angiotensin receptor blockers (ARB) or SGLT2i even if the medication is strongly indicated. A significant number of the expatriates in our cohort are either unemployed or work at extremely low-paying jobs, which forces them into poor housing conditions and consumption of less healthy food. This haunts patients back when they visit the emergency department for any reason and discover that their BP and/or blood sugar is out of control and/or they have advanced CKD. And if such a patient needs hospital admission the financial cost will increase and the prognosis will worsen, especially if a patient needs to start acute dialysis, which has its complications. The other small number of medically insured expatriate patients could follow in the private sector, which gives them access to more laboratory tests and more medications. Greater access to healthcare and higher income might explain the improved glycemic and BP control and better lipid profile for Kuwaiti patients compared to expatriates despite Kuwaitis’ higher BMI.

CKD management has evolved significantly over the past few years with the introduction of SGLT2i. For almost 20 years, RAASi were the gold standard for CKD management, especially when associated with DM, HTN, and/or proteinuria [15, 16]. Yet, less than 50% of our sample received RAASi, even though almost 90% were diagnosed with HTN and reported a mean eGFR of 30 ml/min. This could not be explained by hyperkalemia, since mean potassium level was normal for all subgroups, and this unfortunately is a widespread finding [17]. The first clinical trial of SGLT2i for renal patients was conducted in 2019 and showed significant cardio-renal benefits from empagliflozin when added to RAASi compared to placebo [18]. Since then, many trials have also reported positive outcomes (class effect) of using SGLT2i in different populations, such as diabetics vs. nondiabetics and patients with normal eGFR vs. advanced CKD with eGFR of ≥ 20 ml/min [19–22]. These agents preserve kidney function and reduce albuminuria, and guidelines recommend their use for CKD 3–5 ND. Yet, only 22.6% of our sample (or 27% of Kuwaiti diabetics with eGFR < 60 mL/min1.73m^2^) were prescribed SGLT2i, despite the majority of our sample being eligible according to the guidelines [12]. These findings call for practical steps that ensure a significant increase in the utilization of RAASi and SGLT2i for CKD management in Kuwait. Again, this has been seen in other parts of the world. Similarly, Eberly et al. concluded that SGLT2i prescription between 2015 and 2019 remained low among patients with CKD and heart failure and that patients with low socioeconomic status were less likely to receive an SGLT2i [23]. Since expatriates in Kuwait do not have access to SGLT2i through MoH facilities, SGLT2i would only be covered under private medical insurance or obtained through the private sector at a cost. For RAASi, expatriates can only receive ACEi but not ARB without cost through public hospital pharmacies. Non-steroidal mineralocorticoid antagonists were not yet approved in Kuwait in 2022 when this study was conducted.

Another vital part of CKD management is BP control. Treatment with RAASi has been the cornerstone therapy in HTN associated with CKD. Different HTN management guidelines have placed RAASi among the first three classes of agents to be used, the other two classes are thiazide or thiazide-like diuretics and long-acting dihydropyridine CCB [24–28]. In our sample, we see greater use of centrally acting agents and CCB. A possible explanation to the issue of antihypertensives selections is that many healthcare professionals in the country become anxious when dealing with CKD patients. Depriving CKD patients with eGFR > 30 ml/min/1.73m^2^ with proteinuria and/or history of congestive heart failure, from RAASi for fear of eGFR decline due to the well-known early and reversible hemodynamic effect of RAASi, and fear of hyperkalemia is common practice. In our cohort, potassium levels were well within the normal range (4.6 mmol/L). This practice of greater use of centrally acting agents and NDH CCB persists despite enormous efforts from nephrology and cardiology educators to inform primary care physicians, internists, and junior nephrologists about the reversible hemodynamic fall in eGFR and about handling hyperkalemia. This suggests that it may not be related to knowledge but rather to inertia. Vasodilators and centrally acting agents are add-on therapies in resistant HTN, but they were of high use in our sample possibly due to physicians’ fear of using RAASi, the restricted access to both ARB and long-acting dihydropyridine agents for expatriates, and the belief that thiazide diuretics use is restricted for volume overload only. Ensuring physicians understand the benefits of prescribing RAASi in all patients who have access to it is another area for major improvement in the care of CKD patients in Kuwait.

## Conclusions

CKD is prevalent in Kuwait due to the high prevalence of DM, HTN, and obesity. However, the number of patients under follow-up care in public nephrology clinics is less than what is expected based on predicted prevalence of CKD 3–5 ND the literature. The low number of CKD 3–5 ND under nephrology care is alarming and calls for better education, screening, and referral practices. Although expatriates enter Kuwait in better health, they develop similar comorbidities to Kuwaiti over time. In our cohort, expatriates who are younger and have a higher eGFR experience restricted access to public health services and lower income, leading to a less healthy diet and suboptimal management. These compounding factors result in higher BP, higher HbA1c in diabetics, and worse lipid profile among the expatriate population. Current guidelines encourage the use of RAASi and SGLT2i in CKD management, even for nondiabetics, to reduce adverse cardiorenal outcomes. These agents have not been utilized widely in Kuwaiti patients, and even less in non-Kuwaiti patients who face limited healthcare access. Similarly, RAASi, thiazides, and long-acting dihydropyridine CCB are not used frequently enough as first- or second-line therapies in managing HTN. Major improvement in the care of CKD patients in Kuwait at all levels (primary care physicians, internists, nephrologists) is urgently required.

## Limitations of the study

Our study was limited because of its observational nature and our focus on only nephrology clinics in public hospitals in Kuwait. However, primary care clinics, other specialties, and private nephrology care represent a much smaller percentage of CKD patients given the tendency of such care providers to refer to nephrology early and the prohibitive cost of private care for many individuals. Our findings only represent the CKD situation in Kuwait at a single point in time. However, our large and comprehensive data set allows us to evaluate a diverse population in terms of ethnicity, socioeconomic status, and access to public healthcare.

## Data Availability

No datasets were generated or analysed during the current study.

## Data Sharing

Deidentified participant data, as well as the study protocol, are available from the corresponding author following reasonable request and the publication of this paper.

## Declarations

### Clinical Trial Number

Not applicable. This study is not a trial. It is an observational study.
